# The need to revisit the definition of mesenchymal and adult stem cells based on their functional attributes

**DOI:** 10.1186/s13287-018-0833-1

**Published:** 2018-03-27

**Authors:** Deepa Bhartiya

**Affiliations:** Stem Cell Biology Department, ICMR-National Institute for Research in Reproductive Health, Jehangir Merwanji Street, Parel, Mumbai, 400012 India

**Keywords:** Stem cells, Mesenchymal cells, Very small embryonic-like stem cells, Asymmetric cell division

## Abstract

A debate is ongoing about the ‘stem cell’ status of mesenchymal stem cells (MSCs). This can easily be resolved based on the definition of a stem cell. ‘True’ stem cells are expected to undergo asymmetric cell divisions (ACD) whereby they divide to self-renew and give rise to a slightly bigger, differentiated cell. However, MSCs like any other adult tissue-specific stem cells, including hematopoietic (HSCs), spermatogonial (SSCs) and ovarian (OSCs) stem cells, do not undergo ACD; rather they undergo rapid symmetrical cell divisions. The true stem cells in adult tissues are possibly the pluripotent stem cells termed very small embryonic-like stem cells (VSELs), which were recently shown to undergo ACD to give rise to tissue-specific stem cells ‘progenitors’ (currently termed ‘adult stem cells’) that in turn undergo rapid symmetric cell divisions and clonal expansion (sphere formation with incomplete cytokinesis) followed by differentiation into tissue-specific cell types. MSCs can be cultured from any tissue source and are an excellent source of growth factors/cytokines and thus could provide a niche for proper functioning of the stem/progenitor cells.

## Viewpoint

Arnold Caplan recently discussed the need to rename mesenchymal stem cells (MSCs) as ‘Medical Signalling Cells’ and that MSCs are derived from perivascular cells, the ‘pericytes’ [1]. One should not have the impression that, on transplantation, MSCs will differentiate into multiple cell types/lineages to bring about regeneration. The mechanism of action of transplanted MSCs is distinct, mostly providing paracrine support. Boregowda et al. [2] disagreed with the concept proposed by Caplan and suggested that defining MSCs as stem cells will better define their potential since the stem cell properties and paracrine functions of MSCs are interdependent. MSCs differentiate into osteoblasts, adipocytes and chondrocytes and are also a very good source of growth factors and RNA/protein laden microvesicles (MVs) and thus have huge therapeutic potential. Based on this observation, Boregowda et al. [2] are confused and argue that if MVs derived from MSCs have regenerative potential, then stem/progenitors are not required for regeneration. Recently Ratajczak and Ratajczak [3] discussed the regenerative potential of MVs, which is being tested in various animal models.

But how do these MVs derived from MSCs act and do they preclude a role for stem/progenitor cells in regenerative medicine? I discuss this based on our studies [4, 5] wherein chemoablated mouse testes were regenerated on transplanting MSCs. It is well known that busulphan treatment depletes the adult mouse testes of sperm and germ cells in the seminiferous tubules whereas Sertoli cells survive. We reported that a novel population of pluripotent stem cells, termed very small embryonic-like stem cells (VSELs), survives in the chemoablated testis [4, 5] and similar stem cells were also detected in azoospermic, human testicular biopsies collected from survivors of childhood cancers [6]. We also provided evidence for the first time that the Sertoli cells (somatic niche providing cells for testicular stem cells) are functionally compromised by chemotherapy [4]. Transplanting bone marrow-derived MSCs into the interstitial space (not within the tubules) of chemoablated testis could restore spermatogenesis. Mesenchymal cells aligned as ‘neo-tubules’ and provided paracrine support to the surviving VSELs in the ‘native’ tubules and these endogenous VSELs underwent differentiation into sperm. Microvesicles could also help restore spermatogenesis in chemoablated testis but we would presume that this approach would provide a one-time beneficial effect whereas transplanting MSCs will provide long-term benefit. Several groups have transplanted mesenchymal cells into chemoablated mouse gonads and reported birth of fertile offspring. These studies were recently compiled in a systematic review [7]; however, none of the studies discuss the underlying mechanism that helps regenerate ablated gonads on transplanting MSCs.

Our results show that transplanted mesenchymal cells do not differentiate into gametes but rather provide paracrine support to endogenous VSELs which differentiated into sperm. Both a healthy niche and stem cells are crucial for regeneration to occur.

### Are MSCs stem cells?

**Fig. 1:**
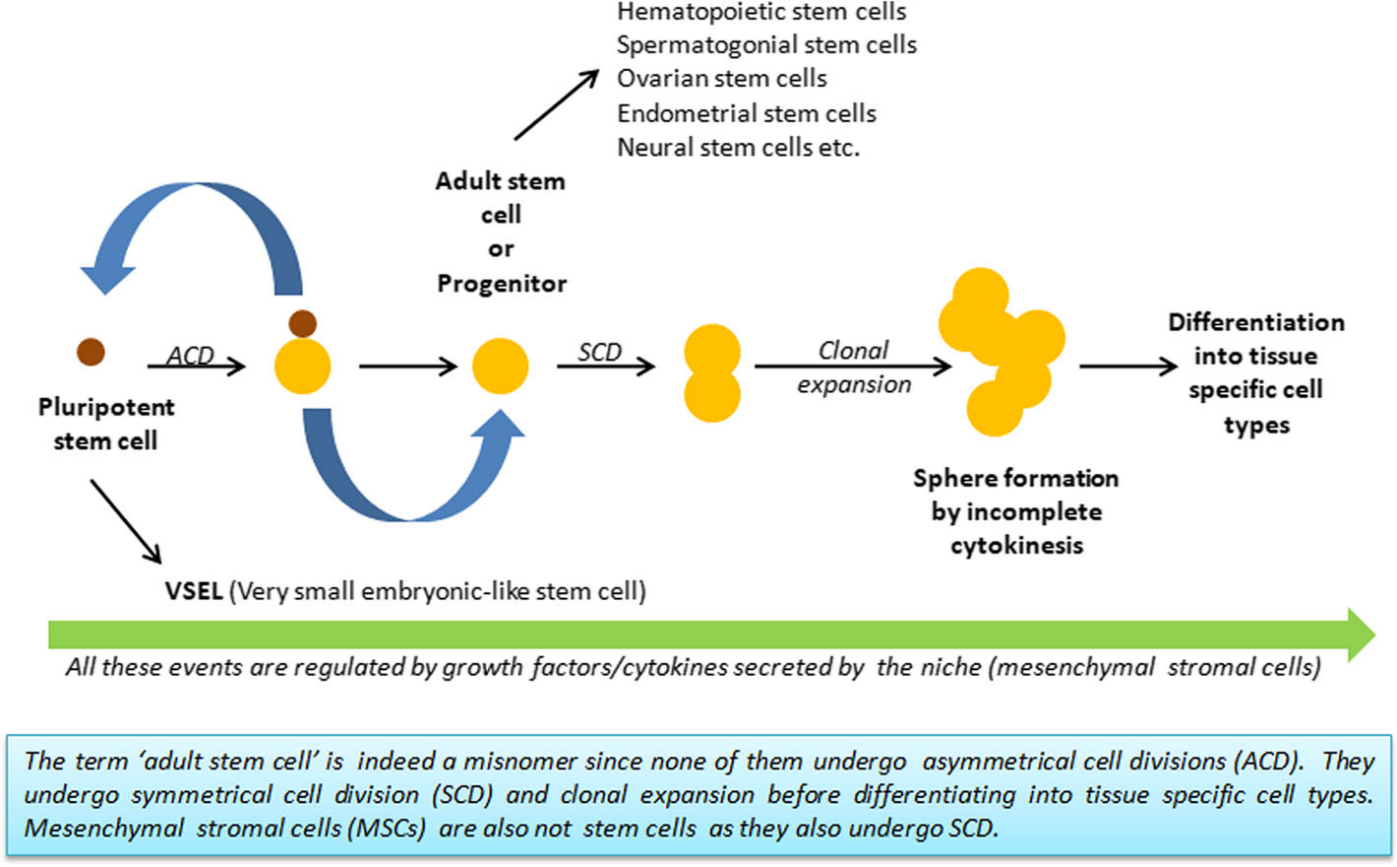
Deciphering stem cell biology in adult tissues. VSELs undergo ACD and are ‘true’ stem cells in adult tissues

A second point of contention is whether MSCs are truly stem cells! True stem cells are expected to undergo asymmetric cell division (ACD) whereby they self-renew and also give rise to differentiated, tissue-committed ‘progenitors’ (https://stemcells.nih.gov/info/2001report/chapter4.htm). We earlier discussed that various adult stem cells like hematopoietic (HSCs), spermatogonial (SSCs), neural (NSCs) and ovarian (OSCs) stem cells etc. are indeed tissue-committed progenitors [8] and labelling them as ‘stem cells’ is a misnomer (Fig. 1). OCT-4 expression and the presence of a sub-population of pluripotent VSELs among MSCs have confused the scientific community [9]. Compared to VSELs with nuclear OCT-4A, adult stem cells (HSCs, SSCs, OSCs and MSCs) express cytoplasmic OCT-4B. This pattern of nuclear and cytoplasmic OCT-4 expression suggests that adult stem cells arise by the differentiation of pluripotent VSELs and have limited plasticity to differentiate only into tissue-specific cell types. Several trials were undertaken across the world using autologous bone marrow mononuclear cells with the hope that the HSCs may regenerate other tissues just like they regenerate ablated bone marrow. However, experience over more than a decade suggests that this has not worked and HSCs fail to regenerate other adult tissues.

Thus, the debate started by Caplan suggesting that ‘MSCs being termed as stem cells is incorrect’ indeed applies to all adult stem cells and is not restricted to only the MSCs. The ‘true’ stem cells in adult tissues are indeed pluripotent VSELs, which exist as a sub-population among MSCs, HSCs, HSCs, NSCs, OSCs, etc., which in turn are ‘multipotent/unipotent’ tissue specific, committed  progenitors (Fig. 1). The pluripotent stem cells have been differently named by various groups and extensively reviewed as VSELs [10, 11] and as MUSE cells amongst MSCs [12] and differentiate into the three germ layers  in both mice and humans.

Data are now emerging that VSELs, being true stem cells, undergo asymmetric cell divisions to self-renew and also give rise to cells with different fates (Fig. 1). Ganguly et al. [13] have shown that, in a dividing doublet, smaller VSELs express nuclear OCT-4A (stem cell marker) whereas slightly bigger cells expresses NUMB (marker for differentiation). Similar ACD along with symmetrical cell division and clonal expansion was recently reported in testis [14] and ovary [15].

To conclude, MSCs are not true stem cells since rather than ACD they undergo rapid and symmetrical cell divisions. MSCs (but no other adult stem cells) have entered the market and several clinical trials are being undertaken using MSCs because they provide paracrine support to endogenous, pluripotent VSELs to function normally and regenerate diseased organs. Our findings that VSELs undergo ACD to give rise to tissue-committed progenitors in various adult tissues [13–15] need to be confirmed by others. It is intriguing that in 2018 we are discussing the definition of stem cells. This understanding becomes crucial in the current scenario when efficacy of adult stem cell therapy to regenerate other organs is being questioned. Also, the field of embryonic and induced pluripotent stem cells has associated safety concerns (genomic and mitochondrial mutations), risk of teratoma formation and immunological issues. It is hoped this Viewpoint article will lead to serious brainstorming and VSELs will be acknowledged as pluripotent, ‘true’ stem cells in adult tissues with regenerative potential.

## Abbreviations

ACD: Asymmetric cell division
MSC: Mesenchymal stem cell
OCT-4: Octamer-binding transcription factor 4
VSEL: Very small embryonic-like stem cells
